# ^1^H-NMR based metabonomic profiling of human esophageal cancer tissue

**DOI:** 10.1186/1476-4598-12-25

**Published:** 2013-04-04

**Authors:** Liang Wang, Jie Chen, Longqi Chen, Pengchi Deng, Qian bu, Pu Xiang, Manli Li, Wenjie Lu, Youzhi Xu, Hongjun Lin, Tianming Wu, Huijuan Wang, Jing Hu, Xiaoni Shao, Xiaobo Cen, Ying-Lan Zhao

**Affiliations:** 1 State Key Laboratory of Biotherapy and Cancer Center, West China Hospital, West China Medical School, Sichuan University, Chengdu 610041, China; 2 Department of thoracic surgery, West China Hospital, West China Medical School, Sichuan University, Chengdu 610041, China; 3 Analytical & Testing Center, Sichuan University, Chengdu 610041, China

**Keywords:** Metabonomic profiling, Human esophageal cancer, ^1^H-NMR

## Abstract

**Background:**

The biomarker identification of human esophageal cancer is critical for its early diagnosis and therapeutic approaches that will significantly improve patient survival. Specially, those that involves in progression of disease would be helpful to mechanism research.

**Methods:**

In the present study, we investigated the distinguishing metabolites in human esophageal cancer tissues (n = 89) and normal esophageal mucosae (n = 26) using a ^1^H nuclear magnetic resonance (^1^H-NMR) based assay, which is a highly sensitive and non-destructive method for biomarker identification in biological systems. Principal component analysis (PCA), partial least squares-discriminant analysis (PLS-DA) and orthogonal partial least-squares-discriminant anlaysis (OPLS-DA) were applied to analyse ^1^H-NMR profiling data to identify potential biomarkers.

**Results:**

The constructed OPLS-DA model achieved an excellent separation of the esophageal cancer tissues and normal mucosae. Excellent separation was obtained between the different stages of esophageal cancer tissues (stage II = 28; stage III = 45 and stage IV = 16) and normal mucosae. A total of 45 metabolites were identified, and 12 of them were closely correlated with the stage of esophageal cancer. The downregulation of glucose, AMP and NAD, upregulation of formate indicated the large energy requirement due to accelerated cell proliferation in esophageal cancer. The increases in acetate, short-chain fatty acid and GABA in esophageal cancer tissue revealed the activation of fatty acids metabolism, which could satisfy the need for cellular membrane formation. Other modified metabolites were involved in choline metabolic pathway, including creatinine, creatine, DMG, DMA and TMA. These 12 metabolites, which are involved in energy, fatty acids and choline metabolism, may be associated with the progression of human esophageal cancer.

**Conclusion:**

Our findings firstly identify the distinguishing metabolites in different stages of esophageal cancer tissues, indicating the attribution of metabolites disturbance to the progression of esophageal cancer. The potential biomarkers provide a promising molecular diagnostic approach for clinical diagnosis of human esophageal cancer and a new direction for the mechanism study.

## Background

Esophageal cancer is one of the most common newly diagnosed cancers and the fourth cause of digestive system cancer mortality in the United States in 2012 [1]. ^1^Esophagectomy is the mainstay of curative treatment for localized esophageal cancer [2]. However, the treatment outcome is far from satisfactory [3-5]. The patients with low-stage cancer have a 45% to 73% chance of survival, and the patients with high-stage tumors of larger size and higher metastatic potential have only a 18% chance of survival within 3 years [6]. The underlying reasons for this disappointingly low survival rate are multifold, including ineffective screening tools and guidelines; cancer detection at an advanced stage, with over 50% of patients with unresectable disease or distant metastasis at the time of presentation; unreliable noninvasive tools to measure complete response to chemoradiotherapy; and limited survival achieved with palliative chemotherapy alone for patients with metastatic or unresectable disease. Therefore, early and accurate diagnosis of esophageal cancer is important for patient survival and improving therapeutic options for different stage of esophageal cancer.

Over the past decades the methods, such as on-endoscopy-based balloon cytology and upper gastrointestinal (GI) endoscopy, have been widely used to improve the diagnosis. However, they have certain limitations including the poor specificity and sensitivity, resulting in detection of the disease at an advanced stage [7]. At the molecular level, numerous studies reporting specific alterations in proteins and genes in esophageal cancer might be useful for the diagnosis, prognosis and treatment of esophageal cancer [8-10]. However, reliable markers, especially at an early and potentially curative stage, are still unknown.

Metabonomics is a systematic approach focusing on the profile of low molecular weight metabolites in cells, tissues, and biofluids [11,12]. It is a powerful tool for analyzing the chemical composition and providing important information on disease process, biochemical functions and drug toxicity [13]. Thus, it has been widely used in disease diagnosis [14,15], biomarker screening [16,17] and safety assessment of chemical [18,19]. Two most powerful and commonly used analytical methods for metabolic fingerprinting are mass spectrometry (MS) and nuclear magnetic resonance (NMR) spectrometry [20,21]. NMR is a non-destructive and non-invasive technique that can provide complete structural analysis of a wide range of organic molecules in complex mixtures [22]. Although a growing number of NMR-based metabonomics aim at finding possible biomarkers of presence and/or grade of different cancers such as prostate cancer [23,24], colorectal cancer [25], brain cancer [26] and breast cancer [27,28], there are only few researches on esophageal cancer [29-31]. Only one report used NMR method to investigate the difference of metabolites in esophageal cancer tissue. Moreover, the number of cancer samples was only 20 ~ 35 in these studies, which may be difficult to provide accurate and comprehensive information of metabolites. Especially, none of these reports systematically investigated the discriminating metabolites involved in the different pathological stages of esophageal cancer.

Multivariate statistical analysis is commonly applied to metabonomic data including the unsupervised (principal component analysis, PCA) and supervised (partial least-squares-discriminant analysis, PLS-DA) methods [32]. In addition, to optimize the separation, thus improving the performance of subsequent multivariate pattern recognition analysis and enhancing the predictive power of the model in NMR-based metabonomic studies [33], orthogonal partial least-squares-discriminant anlaysis, OPLS-DA is carry out to visualize the metabolic alterations between the esophageal cancer tissues and normal esophageal mucosae.

In the present study, we applied ^1^H-NMR to study metabonomic profiling of human esophageal cancer tissues. We identified a total of 45 distinguishing metabolites, 12 of which were modified along with the aggressive process of esophageal cancer. These metabolites are closely associated with the energy supplies, fatty acids and choline metabolic pathways. Our results provide the potential biomarkers for clinical diagnosis for different stages of human esophageal cancer and new insights for the mechanism research. Moreover, this study demonstrates that a NMR–based metabolomics approach is a reliable and sensitive method to study the biochemical mechanism underlying human esophageal cancer.

## Results

### Clinical population

We investigated a total of 115 samples, 89 of which were from primary esophageal cancer and 26 from normal esophageal mucosae. For 26 cases paired samples of cancer tissue and normal tissue were available from the same patient. The clinical information of patients was summarized in Table 1. As listed in Table 1, the patients aged 39–79 years old. The stage of all tissue specimens was determined according with the American Joint Committee on Cancer (AJCC) for esophageal tumors: stage II, 28 patients; stage III, 45 patients; stage IV, 16 patients. Though the tissue specimens of stage I patient was absent because of the limited specimens, it is still worth investigating. 85 cases of cancer samples were esophageal squamous cell carcinoma. All patients were subjected to surgical resection of the primary tumor and lymph nodes. Tumor size, location, lymph node numbers, differentiation status and lymphovascular invasion were also evaluated.

**Table 1:** Clinical information of esophageal cancer patients and normal mucosae

**Clinical features**	**Esophageal cancer**	**Normal control**
Number	89	26
Sex		
Male	82	22
Female	7	4
Age at diagnosis		
Median(years)	61	59.5
Range	39-79	43-70
Histology		
ESCC	85	
Adenocarcinoma	4	
Pathologic grade		
PD	22	
MD	62	
HD	5	
Disease stage		
I	0	
II	28	
III	45	
IV	16	
Lymph node metastasis		
NO	34	
N1	55	

*MD*: poorly differentiated; *PD*: moderately differentiated; *HD*: highly differentiated.

### Metabonomic profiling of samples

**Figure 1:**
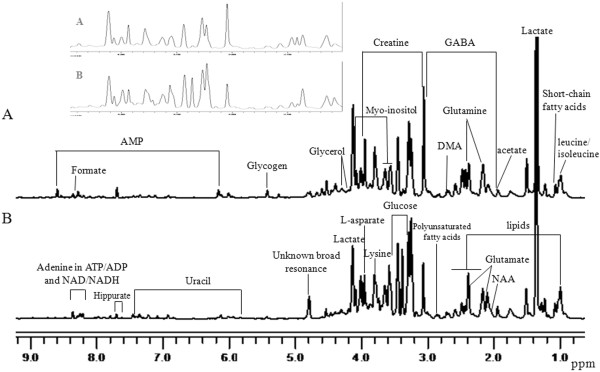
**600.13 MHz CPMG **^**1**^**H-NMR spectra of tissue specimens from esophageal cancer patients and normal mucosae. A**: normal mucosae, **B**: esophageal cancer. The grey region represents the detailed spectra between 2 and 4.5 ppm of normal mucosae and esophageal cancer.

Normal mucosae and esophageal cancer tissue samples underwent extraction, and the aqueous fractions were investigated using NMR. The representative ^1^H-NMR spectra of aqueous phase extracts of normal mucosae and esophageal cancer tissues were showed in Figure 1. The spectra were processed and converted into 419 integral regions of 0.02 ppm width as described in Materials and Methods. The major metabolites in the integrate regions were identified by a comparison with literature data and spectra of standards acquired in Human Metabolome Database. As a result, a series of changes of endogenous metabolite levels were observed in esophageal cancer when compared with the normal mucosa (Figure 1A and 1B). The majority of metabolites were assigned to amino acid, lipid, carbohydrate, organic acid and nucleotide, which are known to be involved in multiple biochemical processes, especially in energy and lipid metabolism [34].

### PR analysis of normal mucosae and esophageal cancers

**Figure 2:**
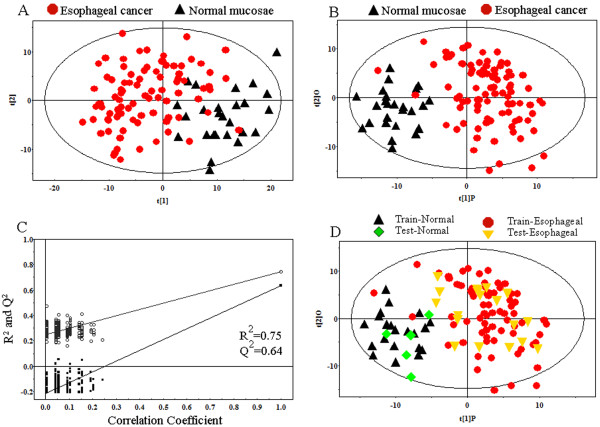
**PR of **^**1**^**H-NMR spectra of tissue specimens. A**, scores plot of PCA model based on 26 cases normal mucosae (black triangles) and 89 cases esophageal cancers (red dots). **B**, scores plot of OPLS-DA model processing based on same samples. **C**, statistical validation of the corresponding PLS-DA model by permutation analysis (200 times). R2 is the explained variance, and Q2 is the predictive ability of the model. **D**, scores plot of OPLS-DA prediction model. 80% of samples (training set, normal mucosae =20, esophageal cancer = 71) were applied to construct the model, and then used it to predict the remaining 20% of samples (testing set, normal mucosae =5, esophageal cancer = 18). Green diamonds represent normal mucosae and yellow inverted triangles represent esophageal cancers.

To optimize the separation of the two groups, we then utilized OPLS-DA to visualize the metabolic difference between the esophageal cancer tissues and normal esophageal mucosae. As the results shown, two groups achieved distinct separation in the scores plot of PC1 and PC2 of OPLS-DA analysis (Figure 2B). Moreover, the corresponding PLS-DA model parameters for the explained variation, R^2^ = 0.75, and the predictive capability, Q^2^ = 0.64, were significantly high, indicating that it is an excellent model suitable for data analysis (Figure 2C). According to the chemical shifts between the two groups (VIP > 1 and p < 0.05), the significantly distinguishing metabolites were identified (Table 2). These metabolites are involved in key metabolic pathways including glycolysis, TCA cycle, urea cycle, pyrimidine metabolism, gut flora metabolism and fatty acids metabolism.

**Table 2:** Summary of different metabolites between esophageal cancers and normal mucosae

**Metabolite**	**Chemical shift(ppm)**	**Esophageal cancer vs Normal control**		
		**VIP**^**a**^	***p*-value**^**b**^	**FC**^**c**^
Amino acid				
Creatine	3.04	2.18	<0.0001	−1.76
	3.94	2.24	<0.0001	−1.78
NAA	2.02	2.07	<0.0001	1.68
	2.6	1.13	<0.0001	1.71
Glycine	3.56	2.07	<0.0001	−1.64
Glutamine	2.14	1.21	<0.0001	−1.34
	2.45	1.93	<0.0001	−1.56
Glutamate	2.05	1.71	<0.0001	1.33
	2.36	1.17	<0.0001	1.23
Valine	0.98	1.70	<0.0001	1.49
Leucine/Isoleucine	0.96	1.61	<0.0001	1.44
4-HPPA	2.45	1.53	<0.0001	−1.60
L-tyrosine	6.93	1.44	<0.0001	1.63
	7.22	1.29	<0.0001	1.51
Phenylacetylglycine	3.68	1.43	<0.0001	−1.86
	7.35	1.06	<0.0001	1.31
	7.42	1.19	<0.0001	1.45
Methionine	2.11	1.21	<0.0001	1.26
	2.16	1.37	<0.0001	1.54
	2.6	1.13	<0.0001	1.71
Creatinine	3.04	2.18	<0.0001	−1.76
	4.05	1.98	<0.0001	−1.57
Phenylalanine	3.12	1.37	<0.0001	1.42
	7.42	1.19	<0.0001	1.45
GABA	1.91	1.23	<0.0001	3.09
	3.01			
Phenylacetyglutamine	7.42	1.19	<0.0001	1.45
Glutamate γ-H	2.36	1.17	<0.0001	1.23
Taurine	3.43	1.02	<0.0001	1.32
L-aspartate	3.92	1.01	0.0451	−1.15
Lipid				
Myo-inositol	3.55	2.11	<0.0001	−1.97
	3.63	2.07	<0.0001	−1.97
	4.07	1.98	<0.0001	−1.57
Unsaturated lipids	2.27	2.08	<0.0001	1.70
Cho	4.05	1.98	<0.0001	−1.57
Short-chain fatty acids	1.04	1.58	<0.0001	1.49
Choline	4.05	1.98	<0.0001	−1.57
	3.51	1.39	<0.0001	−1.45
Phosphocholine	3.22	1.16	<0.0001	1.24
Carbohydrate				
Glucose	3.25		0.026	−1.16
	3.53	2.11	<0.0001	−1.97
Glycoprotein	2	2.08	<0.0001	1.70
Polyol				
Ethanol	3.65	2.07	<0.0001	−1.97
Acetone	2.22	1.28	<0.0001	1.56
Organic acid				
α-ketogultaric acid oxime	2.44	1.53	<0.0001	−1.60
	2.48	1.93	<0.0001	−1.56
Malonate	3.16	1.34	<0.0001	1.46
Acetoacetic acid	2.27	1.27	<0.0001	1.25
	3.43	1.02	<0.0001	1.32
Acetate	1.92	1.23	<0.0001	3.09
Trimethylamine	2.88	1.19	<0.0001	2.19
Formate	8.45	1.01	0.0005	8.79
Nucleotide				
Uracil	5.81	1.82	<0.0001	39.93
	7.54	1.26	<0.0001	2.13
AMP	6.15	1.76	<0.0001	−2.05
	8.67	1.19	<0.0001	−3.10
NAC1	2.1	1.15	<0.0001	1.49
Adenine in ATP/ADP and NAD/NADH	8.25	1.56	<0.0001	1.41
	8.27	1.58	<0.0001	1.75
	8.36	1.45	<0.0001	1.97
NAC2	2.04	1.69	<0.0001	1.68
Cofactors and vitamins				
NAD	8.83	1.65	<0.0001	−2.81
	9.15	1.49	<0.0001	−2.44
	9.35	1.53	<0.0001	−2.43
Inorganic acid				
Acetyl hydrazine	1.96	1.63	<0.0001	2.14
Xenobiotics				
Hippurate	7.55	1.00	0.0005	1.77
	7.64			

^a^Variable importance in the projection (VIP > 1) was obtained from OPLS-DA analysis.

^b^p-value determined from Student's *t*-test.

^c^FC: fold change between esophageal cancers and normal mucosae. Positive sign indicates a higher level in esophageal cancer and a negative value indicates a lower level.

To study the predictive power of the model to unknown samples, we randomly selected 80% of samples (normal mucosae = 20, esophageal cancer = 71) as training set to construct OPLS-DA model, which was used to predict the class membership of the remaining 20% of samples (the ‘testing set’, normal mucosae = 5, esophageal cancer = 18). As shown in Figure 2D, normal mucosae of testing set were correctly located in the region of normal mucosae of training set, and the same results were obtained in esophageal cancers of testing set (R^2^X_cum_ = 0.17, R^2^Y_cum_ = 0.531, Q^2^ Y_cum_ = 0.322). These results show that OPLS-DA model can not only distinguish normal mucosae from esophageal cancers, but also achieve excellent predictive power to the unknown samples.

### PR analysis of normal mucosae and different stages of esophageal cancer

**Figure 3:**
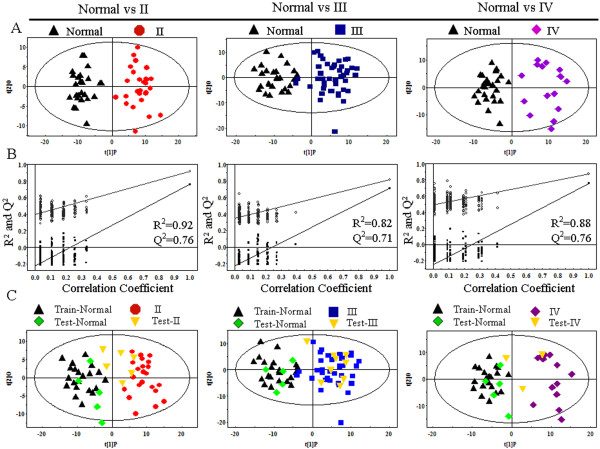
**PR analysis of **^**1**^**H-NMR spectra between different stages of esophageal cancer and normal mucosae. A**, scores plot of OPLS-DA model processing based on each stage and normal mucosae, red dots represent stage II (n = 28); blue boxes represent stage III (n = 45); purple diamonds represent stage IV (n = 16) and black triangles represent normal mucosae (n = 26). **B**, statistical validation of the corresponding PLS-DA model by permutation analysis (200 times). R^2^ and Q^2^ represent the predictive ability to the model. **C**, scores plot of OPLS-DA prediction model. 80% of samples were applied to construct the model, and then used it to predict the remaining 20% of samples (normal mucosae = 5; stage II: n = 5; stage III: n = 9; stage IV: n = 3). Green diamonds represent normal mucosae and yellow inverted triangles represent esophageal cancers.

The differences of metabolites profiling among various stages of esophageal cancer are important for biomarker identification for accurate diagnosis and therapy. The PR analysis showed the metabolites modified, and we then used the OPLS-DA model to investigate the metabolites differentially regulated. As shown in Figure 3A, the scores plot of PC1 and PC2 indicated that all stages (II, III and IV) of esophageal cancer could be clearly separated from normal mucosae. The statistical validations of the corresponding PLS-DA model by permutation analysis were shown in Figure 3B. The parameters for different stages were as follows: stage II: R^2^ = 0.92, Q^2^ = 0.76; stage III: R^2^ = 0.82, Q^2^ = 0.71 and stage IV: R^2^ = 0.88, Q^2^ = 0.76. The panel of 45 metabolites with VIP > 1 and *p* < 0.05 of these three groups were listed in Table 3. The majority metabolites were similar to those of metabolites between normal mucosae and esophagus cancers. Interestingly, we identified dimethylamine (DMA), dimethylglycine (DMG), polyunsaturated fatty acids and histidine, which are associated with the stage of esophageal cancer. DMG significantly decreased in stage III, and the changes of DMA and histidine were significant only in stage IV in comparison to normal mucosae. In addition, polyunsaturated fatty acids were altered significantly in stage II.

**Table 3:** Summary of different metabolites between each stage of esophageal cancers and normal mucosae

**Metabolite**	**Chemical shift (ppm)**	**Normal vs II**			**Normal vs III**			**Normal vs IV**		
		**VIP**^**a**^	***p*-value**^**b**^	**FC**^**c**^	**VIP**^**a**^	***p*-value**^**b**^	**FC**^**c**^	**VIP**^**a**^	***p*-value**^**b**^	**FC**^**c**^
Amino acid										
Creatine	3.04	1.97	<0.0001	-1.71	1.97	<0.0001	-1.70	1.89	<0.0001	-1.91
	3.94	1.92	<0.0001	-1.71	1.90	<0.0001	-1.73	1.89	<0.0001	-1.92
NAA	2.6	2.06	0.001	1.59	1.25	<0.0001	1.84	1.17	0.0003	1.58
	2.02	1.39	<0.0001	1.63	1.90	<0.0001	1.61	1.95	<0.0001	1.81
Glutamate	2.05	1.77	<0.0001	1.34	1.50	<0.0001	1.28	1.77	<0.0001	1.39
	2.36	1.38	<0.0001	1.27	1.11	<0.0001	1.19	1.29	<0.0001	1.26
Glutamine	2.14									
	2.45	1.73	<0.0001	-1.66	1.74	<0.0001	-1.52	1.74	<0.0001	-1.52
Glycine	3.56	1.58	0.0003	-1.48	1.96	<0.0001	-1.66	1.66	<0.0001	-1.71
Creatinine	3.04	1.97	<0.0001	-1.71	1.97	<0.0001	-1.70	1.89	<0.0001	-1.91
	4.05	1.56	0.0005	-1.45	1.85	<0.0001	-1.55	1.74	<0.0001	-1.69
Glutamate γ-H	2.36	1.38	<0.0001	1.27	1.11	<0.0001	1.19	1.29	<0.0001	1.26
Valine	0.98	1.46	0.0008	1.45	1.61	0.0009	1.51	1.45	<0.0001	1.49
Taurine	3.43				1.18	0.0005	1.39	1.39	0.03	1.24
Leucine/Isoleucine	0.96	1.43	<0.001	1.40	1.55	<0.0001	1.45	1.36	<0.0001	1.44
L-tyrosine	6.93	1.22	0.005	1.61	1.41	<0.0001	1.62	1.21	<0.0001	1.66
	7.22	1.11	0.013	1.49	1.27	<0.0001	1.49	1.11	<0.0001	1.54
L-aspartate	3.92				1.16	0.0004	-1.19	1.08	0.0216	-1.15
GABA	1.91	1.58	<0.0003	2.74	1.21	<0.0001	2.88	1.50	<0.0001	3.65
	3.01		0.049	1.15		0.012	1.19	1.11	0.0005	1.29
Methionine	2.11		0.04	1.26	1.11	0.0122	1.22	1.06	0.001	1.34
	2.16	1.56	<0.0001	1.47	1.46	<0.0001	1.57	1.25	<0.0001	1.53
	2.6	1.39	<0.0001	1.59	1.25	<0.0001	1.84	1.17	<0.0001	1.58
Phenylacetyglutamine	7.42	1.01	0.024	1.38	1.16	0.0006	1.43	1.12	0.0005	1.52
Histidine	7.09	1.03	0.027	-2.22	1.30	<0.0001	-3.48	1.99	<0.0001	-2.03
	7.79		0.032	-2.82		0.038	-2.01	1.48	0.0003	-3.93
4-HPPA	2.45	1.16	0.0116	-1.61	1.37	<0.0001	-1.65	1.01	0.0026	-1.53
Dimethylglycine	3.72				1.03	0.0016	-1.50			
Phenylacetylglycine	3.68									
	7.42	1.01	0.024	1.38	1.16	0.0006	1.43	1.12	0.0005	1.52
	7.35	1.3	0.0381	1.28	1.28	0.0038	1.2	1.23	0.0024	1.35
Lipid										
Polyunsaturated fatty acids	2.84		0.0113	1.25	1.02	0.0023	1.29	1.03	0.033	1.24
Unsaturated lipids	2.27	1.04	0.01	2.23	1.17	0.0002	2.24	1.19	0.0002	2.09
Short-chain fatty acids	1.04	1.39	0.001	1.41	1.49	<0.0001	1.48	1.53	<0.0001	1.55
Phosphocholine	3.22	1.56	0.0002	1.29	1.07	0.03	1.11	1.25	<0.0001	1.05
Myo-Inositol	3.55	1.81	<0.0001	-1.76	2.10	<0.0001	-2.01	1.71	<0.0001	-2.04
	3.63	1.71	<0.0001	-1.62	2.01	<0.0001	-1.81	1.68	<0.0001	-1.86
	4.07	1.56	0.0005	-1.45	1.85	<0.0001	-1.55	1.74	<0.0001	-1.69
Cho	4.05	1.56	0.0005	-1.45	1.85	<0.0001	-1.55	1.74	<0.0001	-1.69
Choline	3.51	1.47	0.001	-1.43	1.63	<0.0001	-1.53		0.006	-1.34
	4.05	1.56	0.0005	-1.45	1.85	<0.0001	-1.55	1.74	<0.0001	-1.69
Carbohydrate										
Glycoprotein	2	2.00	<0.0001	1.64	1.89	<0.0001	1.66	1.95	<0.0001	1.81
Glucose	3.25							1.16	0.0005	-1.34
	3.53	1.81	<0.0001	-1.76	2.10	<0.0001	-2.01	1.71	<0.0001	-2.04
Polyol										
Acetone	2.22	1.47	0.0007	1.38	1.32	<0.0001	1.49	1.47	<0.0001	1.79
Ethanol	3.65	1.71	<0.0001	-1.62	2.01	<0.0001	-1.81	1.68	<0.0001	-1.86
Organic acid										
α-ketogultaric acid oxime	2.44	1.16	0.01	-1.61	1.37	<0.0001	-1.65	1.01	0.002	-1.53
	2.48	1.74	<0.0001	-1.52	1.74	<0.0001	-1.52	1.73	<0.0001	-1.66
Malonate	3.16	1.11	0.013	1.35	1.31	<0.0001	1.45	1.38	<0.0001	1.53
Acetate	1.92	1.58	<0.0003	2.74	1.21	<0.0001	2.88	1.50	<0.0001	3.65
Acetoacetic acid	2.27	1.04	0.01	1.22	1.17	0.0002	1.25	1.19	0.0002	1.28
	3.43				1.18	0.0005	1.39	1.39	0.03	1.24
Formate	8.45	1.44	<0.001	13.51	1.11	<0.001	14.84	1.31	<0.0001	21.48
Trimethylamine	2.88	1.23	<0.0001	2.09	1.41	<0.0001	2.24	1.28	0.005	2.23
Dimethylamine	2.73							1.31	0.0057	1.67
Nucleotide										
NAC2	2.04	1.69	<0.0001	1.35	1.50	<0.0001	1.31	1.65	<0.0001	1.44
NAC1	2.1		0.0495	1.26	1.11	0.0122	1.22	1.06	0.0011	1.34
AMP	6.15	1.50	<0.0001	-1.76	1.72	<0.0001	-2.10	1.60	<0.0001	-2.16
	8.67				1.20	<0.0001	-3.18	1.12	0.0007	-3.84
Adenine in ATP/ADP and NAD/NADH	8.36	1.33	<0.0001	2.02	1.45	<0.0001	2.00	1.14	0.011	1.78
	8.27	1.52	<0.0001	1.44	1.51	<0.0001	1.41	1.59	<0.0002	1.38
	8.25	1.46	<0.0001	1.44	1.51	<0.0001	1.41	1.59	<0.0002	1.38
Uracil	5.81	1.75	<0.0001	43.30	1.83	<0.0001	37.49	1.84	<0.0001	41.03
	7.54	1.31	0.003	2.12	1.28	<0.0001	2.06	1.26	<0.0001	2.24
Cofactors and vitamins										
NAD	8.83	1.56	0.0004	-2.28	1.53	<0.0001	-2.44	1.81	<0.0001	-4.55
	9.15	1.54	0.0005	-2.09	1.41	<0.0001	-2.22	1.65	<0.0001	-3.28
	9.35	1.52	0.0005	-2.12	1.39	<0.0001	-2.09	1.78	<0.0001	-3.65
Inorganic acid										
Acetyl hydrazine	1.96	1.99	<0.0001	2.02	1.62	<0.0001	2.03	1.75	<0.0001	2.40
Xenobiotics										
Hippurate	7.55	1.08	0.0171	1.78		0.0027	1.75	1.13	0.0019	1.81
	7.64	1.21	0.0079	1.12	1.28	0.0351	1.38	1.03	0.0006	1.16

^a^Variable importance in the projection (VIP > 1) was obtained from OPLS-DA analysis.

^b^*p*-value determined from Student's *t*-test.

^c^FC: fold change between esophageal cancers and normal mucosae. Positive sign indicates a higher level in esophageal cancer and a negative value indicates a lower level.

Finally, we randomly used 80% of samples to construct OPLS-DA model, and then detected the predictive power to the remaining 20% of samples. As shown in Figure 3C, the majority samples of testing set were correctly classified as esophageal cancer and normal mucosae. In the scores plot of stage II and normal mucosae (R^2^X_cum_ = 0.287, R^2^Y_cum_ = 0.895, Q^2^Y_cum_ = 0.725), the green diamond which represented normal mucosae of testing set were correctly located in the region of normal mucosae, and most of the stage II samples of testing set located in the region of stage II. These results indicate that esophageal cancer of stage II are correctly discriminated from normal mucosae. However, there were 2 samples of stage II located in the edge of the two training set clusters. One possible reason might be that the changes of the metabolites were not significant in the earlier stage. For example, DMA was only remarkably increased in stage IV. The scores plot of stage III, IV (stage III: R^2^X_cum_ = 0.276, R^2^Y_cum_ = 0.798, Q^2^Y_cum_ = 0.671; stage IV: R^2^X_cum_ = 0.296, R^2^Y_cum_ = 0.912, Q^2^Y_cum_ = 0.765) were illustrated in Figure 3C Almost all testing set were correctly located in the corresponding region except for one testing set of stage IV. These results indicate that metabonomics difference could be used for the grading of esophageal cancer.

### Trending markers

**Figure 4:**
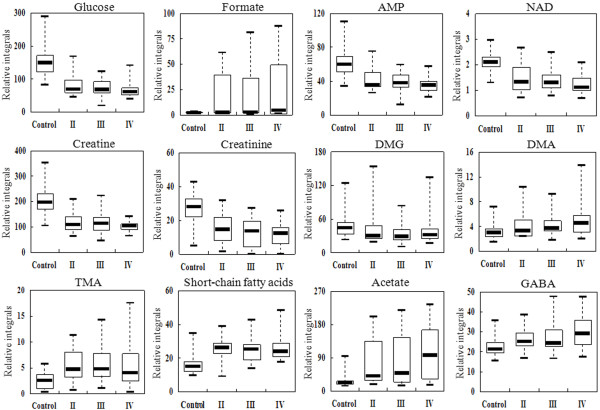
**Box-and-whisker plots illustrated progressive changes of the metabolites among different stages of esophageal cancer relative to normal mucosae.** Horizontal line in the middle portion of the box, median; bottom and top boundaries of boxes, lower and upper quartile; whiskers, 5th and 95th percentiles; open circles, outliers.

To further study which biomarkers are mainly responsible for the pathological process of esophageal cancer disease, we used box-and-whisker plots to clarify the relative altered levels of those identified 45 metabolites, and obtained 12 representative metabolites among normal control mucosae and esophageal cancer at different stages (Figure 4). Interestingly, these metabolites included glucose, formate, AMP, NAD, creatine, creatinine, DMG, DMA, trimethylamine (TMA), short-chain fatty acids, acetate and GABA, which are mainly involved in energy, fatty acids and choline metabolic pathways.

Glucose, the main source of energy metabolism and precursors for biosynthesis of macromolecules in cells [35], was decreased along with the progression of esophageal cancers when compared with normal mucosae. Formate, the product of sugar utilization of Enterococcus casseliflavus [36], was significantly increased along with the progression; moreover, it upregulated 21.48 folds in stage IV compared to normal mucosae. The AMP, which can be transformed to ATP as energy donor, and NAD, an important coenzyme, were downregulated in esophageal cancer tissues, suggesting a great quantity of energy consumption due to accelerated cell proliferation in esophageal cancer. The downregulation of creatine, creatinine and DMG, and the elevation of DMA and TMA along with the stages of esophageal cancer indicate the disturbance of choline metabolism. Acetate and GABA, two intermediates in the synthesis of fatty acids, were significantly elevated along with the progression, and both of them upregulated 3.65 folds in stage IV compared to normal mucosae. In addition, the upregulation of short-chain fatty acids, a kind of fatty acid materials, indicated a highly activated fatty acids metabolism in esophageal cancer.

## Discussion

In the present study, we discriminated 89 esophageal cancer tissues from 26 normal mucosae using an OPLS-DA model, and analyzed the metabolites difference between each stage of esophageal cancer and normal mucosae to identify the potential biomarkers involved in the development of esophageal cancer. Forty-five of distinguishing metabolites were identified and 12 of them, including glucose, formate, AMP, NAD, creatine, creatinine, DMG, DMA, TMA, short-chain fatty acids, acetate and GABA, were significantly changed along with the progression of esophageal cancer. Though there are several reports showing the metabolic profiling of esophageal cancer, to the best of our knowledge, the present study is the first to show that some specific metabolites are modified along with the stage of esophageal cancer. Remarkably, the highly activated fatty acids metabolism, increased energy supplies and disturbance of choline metabolism are mainly responsible for the process of pathological development of esophageal cancer.

**Figure 5:**
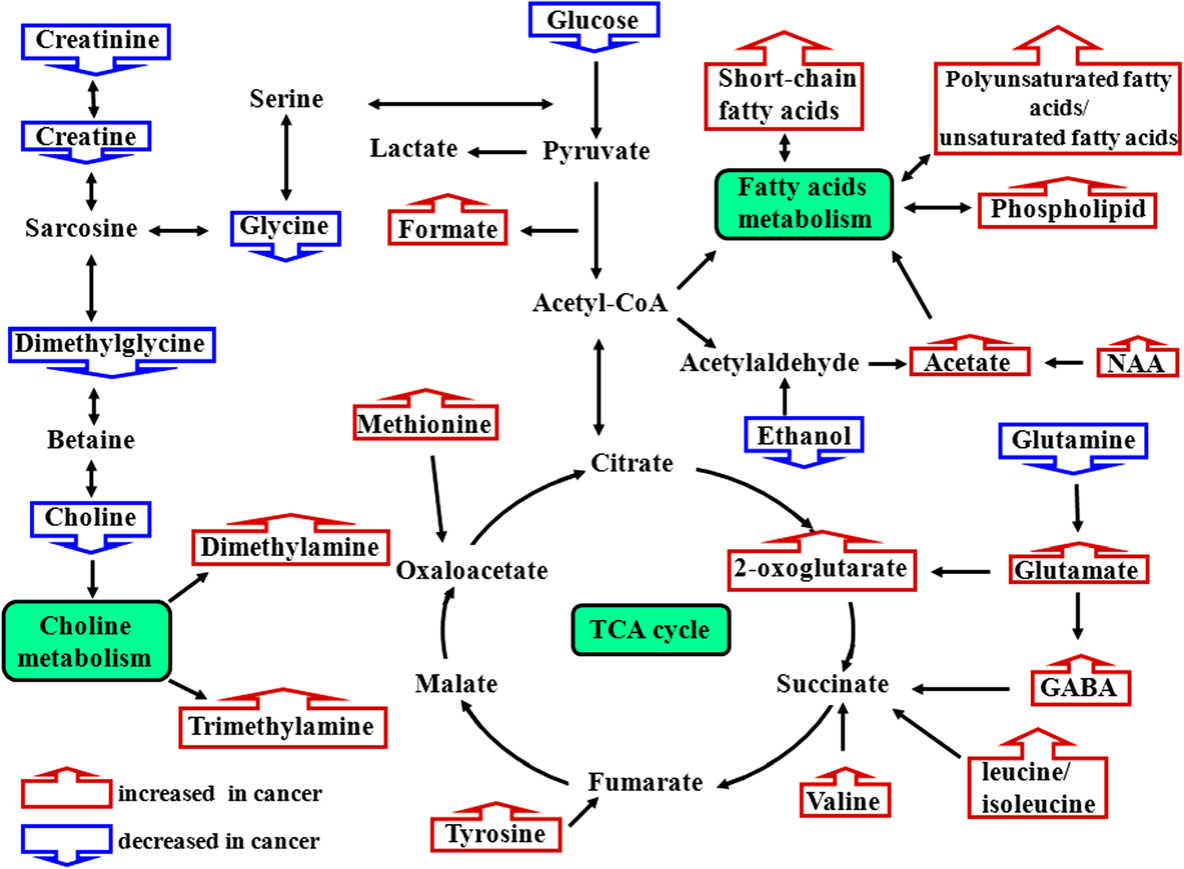
**Altered metabolic pathways for the most relevant distinguishing metabolites between esophageal cancers and normal mucosae.** Blue boxes indicated metabolites that were up-regulated in esophageal cancers, while red boxes indicated metabolites that were down-regulated.

Identifying the related metabolic pathways of distinguishing metabolites is very important for understanding the biochemical alterations during neoplastic occurrence and development. In order to enhance the information obtained from global metabonomic profiling of esophageal cancer, the human metabolome database and the Kyoto encyclopedia of genes and genomes (KEGG) were utilized to map the marker metabolites with regards to the human metabolic pathways. The findings about the key sets of marker metabolites, related metabolic pathways are summarized in Figure 5. These distinguishing metabolites are involved in detailed metabolic pathway, including fatty acids metabolism (polyunsaturated lipids, short-chain fatty acids, phospholipid, NAA and acetate, GABA), choline metabolism (choline, DMA, DMG, TMA creatine and creatinine), amino acid metabolism (glycine, L-aspartate, glutamine, valine, leucine/isoleucine, methionine and tyrosine), glycolysis (glucose), glutaminolysis (glutamine and glutamate) and tricarboxylic acid cycle (2-oxoglutarate). These results indicate that several specific metabolic pathways are disturbed in esophageal cancer tissue and, particularly, fatty acids metabolism, energy supplies and choline metabolic pathways are involved in the progression of esophageal cancer.

In the process of cancer development, cancer cells increase and alter the metabolism of major nutrient, glucose via glycolysis to meet the high-energy demand under hypoxic conditions [37,38]. In 1920s, Otto Warburg first discovered that cancer cells prefer to metabolize glucose through glycolysis to generate ATP instead of oxidative phosphorylation even in presence of ample oxygen [39]. One molecule of glucose only generates 2 molecules of ATP through glucolysis. This process is a less efficient pathway compared with oxidative phosphorylation which generated ~36 molecules of ATP [40]. Therefore, plently of glucose was consumpted, resulting in glucose reduction. Our results showed that the level of glucose was decreased in esophageal cancer tissue, which is similar to the previous studies that decreased glucose is detected in human colorectal cancer, cervical cancer and hepatoma [41-43]. Moreover, formate, the product of glucose utilization, was significantly increased along with the progression. Remarkably, it reached to the 21 fold in the stage IV in comparison to normal mucosae. Thus, it should be considered as a potential biomarker involved in the progression of human esophageal cancer. Besides glycolysis, increased glutaminolysis is recognized as a vital metabolism pathway of cancer cells to meet the high-energy demand under hypoxic conditions. In cancer cells, glutamine is converted to glutamate by mitochondrial glutaminase. Glutamate is an important energy sourse via anaplerotic input into the the tricarboxylic acid (TCA) cycle after conversion to α-ketoglutarate. The decrease of glutamine observed in esophageal cancer could satisfy the production of energy. After conversion to glutamate and ketoglutarate, the final major fate of glutamine is the oxidation of its carbon backbone in the mitochondria, leading to energy production. Glutaminolysis contributes to production of mitochondrial NADH, which is used to support ATP production by oxidative phosphorylation. In the present study, the decreased AMP in esophageal cancer tissue suggests a rapid energy transformation due to a great demand of ATP synthesis.

Besides supporting ATP production, glutamine also contributes to the biosynthesis of lipids and nucleic acids, and regulation of redox homeostasis [44,45]. The catabolism of glutamine is initiated by glutaminolysis [46]. In glutaminolysis, malate is convered into pyruvate or carboxylation is reduced to produce acetyl-CoA. Both catabolism could be useful for maintaining lipogenesis [47,48]. In proliferating cancer cells, the lipogenesis is especially needed for the formation of cellular membranes [39,49]. Therefore, another reasonable explanation for the decreased glutamine observed in present study might be that glutamine is used for maintaining the formation of cellular membranes in esophageal cancer tissues. In the cancer cell proliferation, besides glutamine, the fatty acids are required for the membrane lipids synthesis due to accelerated cell proliferation. In present study, the family of fatty acid materials, short-chain fatty acids, phospholipid, and polyunsaturated fatty acids were upregulated, indicating the activation of fatty acids metabolism. In addition, previous studies reported that cancer tissue can ultilize GABA to produce propylene glycol, a precursor of pyruvate derived from glycine [50]. Therefore, GABA upregulation in esophageal cancer tissue could be used as building blocks for biosynthesis of cellular membranes. Importantly, GABA increased dramatically along with the progression of esophageal cancer. Therefore, GABA might play a role in the deterioration or matastasis of esophageal cancer. NAA, a free amino acid, synthesized from L-asparate and acetyl-CoA, serves as a source of acetate for lipid and myelin synthesis [51]. We found that both NAA and acetate were significantly increased in esophageal cancer tissue, but L-asparate was decreased. These results are consistent with other findings in ovarian cancer [52]. However, in the previous metabonomics research on esophageal cancer, none of the paper reported the identification of NAA and acetate. The possible reason is that NMR spectroscopy is a method that no selection to metabolites and thus all possible variables could be detected in which no prior information about metabolites is known or the higher sensitivity compared to ^1^H MAS-NMR [53].

Choline and its derivatives represent important constituents in phospholipid metabolism of cell membranes and have been previously identified as markers of cellular proliferation. Choline is degraded through two pathways. The one is to form creatinine via DMG, and the other is converted to methylamine [54]. In the present study, the levels of creatinine, creatine, glycine, DMG and choline significantly decreased in esophageal cancer tissues. Meanwhile, DMA and TMA, the products of choline metabolism, remarkable increased [55]. Though these two products are widely regarded as nontoxic substance, their potential to form the carcinogen NDMA attaches great clinical interest [56]. Our results indicate the disturbance of choline metabolism in esophageal cancer tissue, which is supported by previous findings that choline and its metabolites allow a distinct differentiation in human oral cancer [32]. The elevation of choline metabolites in esophageal caner may be resulted from the metabolism of membrane phospholipids due to accelerated cell proliferation. The levels of DMG, creatinine, creatine, DMA and TMA were altered along with development of esophageal cancer, indicating that they might be the potential biomarkers for diagnosis of esophageal cancer.

## Conclusions

Overall, our findings confirm a distinct tissue metabolic profile of esophageal cancer patients characterized by altered levels of 45 metabolites mainly involved in fatty acids metabolism, energy supplies and choline metabolism. Particularly, a panel of 12 metabolite biomarkers was changed along with the development of esophageal cancer and might be related to the occurrence and aggression of this cancer. Our study highlights the significance of the distinct tissue metabolic profile of esophageal cancer. These 12 gradient biomarkers provide not only a new insight for the establishment of improved clinical biomarkers for esophageal cancer detection, but also potential information for mechanism study of esophageal cancer progression. Further investigation is needed to validate these initial findings in much larger samples and the related mechanism underlying the progression of esophageal cancer.

## Methods

### Chemicals

Deuterium oxide (99.8% D) was purchased from NORELL (Landisville, USA). Trimethylsilylpropionic acid-d4 sodium salt (TSP) was purchased from Sigma Aldrich (St. Louis, MO). HPLC-grade methanol and chloroform were purchased from Fisher Scientific (Fairlawn, NJ, USA). Deionized water was obtained from an EASYpure II UV water purification system (Barnstead International, Dubuque, IA). All of the chemicals employed in this study were of analytic pure and culture grade.

### Sample collection

The protocol of the present study was approved by the Ethics Committee of West China Hospital of Sichuan University. The informed consents were obtained from all patients.

In total, 115 case of surgical specimen came from eighty-nine esophageal cancer patients treated during 2010 to 2011 at West China Hospital of Sichuan University. Among them, 52 cases belonged to the matched tumor and normal mucosae, which were taken at least 5–10 cm away from the edges of a tumor from the same patient (n = 26). The patients enrolled in this research did not receive any neoadjuvant chemotherapy or radiation therapy prior to esophagectomy. Fresh tumor tissues or corresponding normal esophageal mucosae were immediately frozen in liquid nitrogen after dissection, then stored at −80 °C until processing. Tumor specimens were carefully microdissected to ensure at least 90% of the analyzed tissue contained cancer cells. The clinical diagnosis, tumor stage, histology differentiation and resection margin were determined by routine histopathological examination of H & E stained specimens by a blinded pathologist.

### Sample Preparation

The 200–500 mg of frozen tissue samples were weighed and suspended in bidistilled water containing methanol (4 ml per gram of tissue). The samples were homogenized with 20 strokes at 800 rpm, and 2 ml/g chloroform was added and homogenization was repeated. Then, the suspension was mixed with 2 ml/g chloroform and 2 ml/g bidistilled water, and leaved on ice for 30 min, followed by centrifugation at 4000 g for 30 min. This procedure separated suspension to three phases, including a water phase containing methanol at the top, a denatured proteins phase in the middle, and a lipid phase at the bottom. The upper phase (aqueous phase) of each sample were collected and evaporated to dryness under a nitrogen gas stream. The residue was reconstituted with 580 μl of D_2_O containing 0.01 mg/ml sodium (3-trimethylsilyl)-2,2,3, 3-tetradeuteriopropionate (TSP) and 30 μmol/L phosphate buffer solution (PBS, pH = 7.4). The D_2_O and TSP provided the deuterium lock signal for the NMR spectrometer and the chemical shift reference (δ0.0), respectively. After centrifuged at 12,000 g for 5 min, the supernatant was transferred into a 5-mm NMR tube for NMR spectroscopy [53].

### ^1^H-NMR Measurements

All samples were detected by ^1^H-NMR spectroscopy at 600.13 MHz using a Bruker Avance II 600 spectrometer operating (Bruker Biospin, Germany) at 300 K. A one-dimensional spectrum was acquired by using a standard (1D) Carr-Purcell-Meiboom-Gill (CPMG) pulse sequence to suppress the water signal with a relaxation delay of 5 sec. Sixty-four free induction decays (FIDs) were collected into 64 K data points with a spectral width of 12,335.5-Hz spectral, an acquisition time of 2.66 sec, and a total pulse recycle delay of 7.66 sec. The FIDs were weighted by a Gaussian function with line-broadening factor 20.3 Hz, Gaussian maximum position 0.1, prior to Fourier transformation [57].

### ^1^H-NMR spectral data processing

The raw NMR data (FIDs) was been manually Fourier transformed for obtaining NMR spectroscopy in MestRe-c2.3 software to reduce the complexity of the NMR data and facilitate the pattern recognition (http://mestre-c-lite.findmysoft.com/download/). After phase adjustment and baseline correction, the spectrum was divided into 419 segments ranging from 9.5 to 0.5 ppm, with equal width of each region (0.02 ppm). The region 5.2–4.6 ppm was removed for excluding the effect of imperfect water suppression. Moreover, the integrated data were normalized before pattern recognition analysis to eliminate the dilution or bulk mass differences among samples due to the different weight of tissue.

### Pattern recognition (PR) analysis

For pattern recognition, the reduced and normalized NMR spectral data were imported into SIMCA-P (version 11, Umetrics AB) for analysis. PCA, the unsupervised PR method, was initially applied to analyze the NMR spectral data to separate the tumor samples from the normal samples. PLS-DA and OPLS-DA, the supervised PR method, were subsequently used to improve the separation and the data filtering method. The PLS-DA models were validated by a permutation analysis (200 times). The default 7-round cross-validation was applied with 1/seventh of the samples being excluded from the mathematical model in each round, in order to guard against overfitting. The variable importance in the projection (VIP) values of all peaks from OPLS-DA models was taken as a coefficient for peak selection, and these variables with VIP > 1 was considered relevant for group discrimination [58]. In addition to the multivariate statistical analysis method, unpaired Student’s *t*-test (*p* < 0.05) to the chemical shifts was also used to the significance of each metabolite. Only both VIP > 1 of multivariate and *p* < 0.05 of univariate statistical significance were identified distinguishing metabolites. Metabolites of corresponding chemical shift were identified according to the previous literatures and the Human Metabolome Database (http://www.hmdb.ca/), a web-based bioinformatic/cheminformatic resource with detailed information about metabolites and metabolic enzymes.

## Abbreviations

1H-NMR: ^1^H nuclear magnetic resonance; PCA: Principal component analysis; PLS-DA: Partial least squares-discriminant analysis; OPLS-DA: Orthogonal partial least-squares-discriminant anlaysis; MS: Mass spectrometry; PC: Principal components; VIP: Variable importance; DMA: Dimethylamine; DMG: Dimethylglycine; TMA: Trimethylamine; AMP: Adenosine monophosphate; ATP: Adenosine triphosphate; NAD: Nicotinamide adenine dinucleotide; GABA: Gamma-aminobutyrate; TCA: Tricarboxylic acid; NAA: N-acetylaspartate; NDMA: N-nitrosodimethylamine.

## Competing interests

The authors declare that they have no competing interests.

## Authors’ contributions

LW, JC-Draft the paper, processed, analyzed the 1H-NMR data, performed the multivariate analysis and also classification of the data. LC, PX, ML WL-were involved in the collection and verification of tissue samples and clinical details. PD-mainly took part in the detection of samples. YZ, HJ-assisted the writing of the paper and the interpretation of its results. TW, HW, JH, XS-were mainly responsible for the extraction of the metabolites and its dryness. YZ-conceived and designed the study and assisted in the interpretation of results. All authors have read and approved the contents.
